# Formulation, characterization of glucosamine loaded transfersomes and in vivo evaluation using papain induced arthritis model

**DOI:** 10.1038/s41598-022-23103-1

**Published:** 2022-11-17

**Authors:** Muhammad Shahzad Rasheed, Sameen Fatima Ansari, Iram Shahzadi

**Affiliations:** Riphah Institute of Pharmaceutical Sciences, Riphah International University, 7th Avenue, Sector G-7/4, Islamabad, Pakistan

**Keywords:** Diseases, Medical research, Nanoscience and technology

## Abstract

The aim of current study was to develop the transdermal transfersomes of glucosamine for better drug delivery. Stretch ability and plasticity of transfersomes membranes mitigate the risk of vesicle rupture in the skin and allows the drug carrying transfersome to pass through the epidermis following the natural water gradient. Transdermal delivery of Glucosamine has an advantage over oral route, having greater local concentration and fewer systemic effects. Thin Film Rotary method was use to prepare transfersomes, and characterization was carried out physio-chemically using electron microscopic studies, zeta potential evaluation, entrapment efficiency studies. To add on in the stability, development of a secondary topical vehicle using Carbopol 940 was carried out to enhance the shelf life of transfersomes. Furthermore, in vivo studies on rabbits were also carried out using the papain induced arthritis model to support the effectiveness of treatment. The radiology studies of knee joint of rabbits proved the effectiveness of glucosamine loaded transfersomes in healing the osteoarthritis with the blood plasma analysis remain unaltered. In vitro characterization showed the successful development of nano-deformable entities with good entrapment efficiency but with little stability, therefore modified into a gel. In a nut shell this modified new dosage from can be best alternative to other conventional options that owe lot of demerits.

## Introduction

Osteoarthritis a joint disease is the important cause of disability and immobilization among older peoples. Articular cartilage allow the free, friction free movement and smooth, white material that cover the end of bones, where the bones joint each other^1^. Usually 2 to 4 mm thick and is free of any nerve or blood vessel. Its composition include dense matrix containing specialized cells chondrocytes along with water, collagen, proteoglycans, and other non-collagenous proteins and glycoproteins to the lesser amount. Chondrocytes plays a vital role in the development and repair of cartilage and maintenance of matrix in which they are embedded. Chondrocytes originate from mesenchymal stem cells and making up to 2% of the total volume of articular cartilage^2^.

80% of the cartilage weighs water. Articular cartilage is separated by a synovial fluid composed of hyaluronic acid and lubricin, proteinases, and collagenases. This fluid is secreted by inner membrane of synovial joints and contains specialized cell synoviocytes.

Osteoarthritis generally involves the damage of articular cartilage and rubbing of bones ends causing pain and stiffness. The diagnosis is based on radiological visualization of joint and the cartilage integrity^3^.

Osteoarthritis can be diagnosed by three ways which are: Pathologically, Radiographically and Clinically. Most common method and simplest method if the visualization of radiological study. The visual inspection of the x-rays can give information about the any damage in the cartilage and any progression of the disease^4^.

Transfersomes are novel drug carriers or a drug delivery technology, highly deformable nature having ability to carry and transport large molecules across skin. Transfersomes are ultra-deformable nano vesicles or ultra-deformable liposomes, composed of phospholipids and edge activators such as Tween 80 or Span 80 that enable extreme flexibility, plasticity of the vesicle shape and lead to generation of elasticity^5^.

This carrier system is composed of at least one amphipathic entity (such as phospholipids like Phosphatidylcholine) which in the presence of aqueous solvents modify and adopt into lipid bilayer that closes into a simple lipid vesicle. Addition of one or a justified combination of bilayer softening components (such as a biocompatible edge activator or a surfactant or an amphiphile drug) lipid bilayer flexibility, deformability and permeability are increased^6^. The resulting nano deformable transfersome vesicles are capable to adapt its shape to requirement easily and rapidly, thus making the transfersomes different from similar morphology nano particles primarily by its flexible, easy adjustable artificial membrane^7^. Transfersomes have inner hydrophilic region for the entrapment of hydrophilic drug while hydrophobic region for water insoluble drug lying with the lipid layer.

The original composition of transfersome include phospholipids such as phosphatidylcholine or soya phosphatidyl choline and edge activators to bring elasticity^8^. Phospholipids are responsible for the formation of membrane of vesicle or transfersome. Polysorbate 80, Polysorbate 60, Span 80, Sodium Cholate etc. edge activators can be used in the formulation of transfersomes. These Edge activators are bilayer softening component, and responsible to bring flexibility and enhance permeability of vesicles^9^. Edge activators and surface charge thereof play a vital role in ensuring the stability of transfersome and achieving the desired deformability. Type and concentration greatly affect the physiochemical properties of transfersomes. Transfersomes made with cationic edge activators show better transdermal transportation in some cases and in some instances non-ionic transfersome are better transdermal drug carriers^10^.

Transfersome are drug mover system and work based upon two main factors: (a) High deformability (elasticity) of the vesicle bilayer and the (b) Osmotic gradient across the skin^11^.

Edge activator help to generate trans-epidermal osmotic gradient and squeezes into the stratum corneum, carries the drug across the skin. The difference in water content across the epidermis and stratum develops the osmotic gradient leading to ease the movement of transfersomes from higher to lower gradient and penetration of transfersomes carrying the drug across the skin^12^. The skin barriers are optimally overcome by the flexibility and deformability of the transfersomal vesicles, which can be harmonized by the relative ratio of surfactant.

The stability is assessed by the zeta potential. Zeta Potential is a measurement of the surface charge on the suspended particles. Charge on the particles generate repulsive forces and avoid particle aggregation. Particles with a zeta potential greater than ± 30 mV are considered to be stable. Stability of transfersome are usually overcome by making a secondary gel^13^. The formation of secondary gel enhances the stability and shelf life of transfersomal vesicles and also ease in application.

Glucosamine (C_6_H_13_NO_5_) is an amino sugar and a precursor in the biochemical synthesis of glycosylated proteins and lipids. Glucosamine is part of the structure of the polysaccharides, chitosan, and chitin. Glucosamine is one of the most abundant monosaccharides. It is produced commercially by the hydrolysis of crustacean exoskeletons or, less commonly, by fermentation of a grain such as corn or wheat^14^.

Glucosamine sulphate is a precursor for glycosaminoglycans and glycosaminoglycans are a major component of cartilage, research has focused on the potential for supplemental glucosamine to beneficially influence cartilage structure and alleviate arthritis.

Studies have been conducted to evaluate the effectiveness and usefulness of glucosamine in treatment of Osteoarthritis, and many researchers in the past showed that it is equally or even better effective than NSAIDs, although its onset of action is slower. It is the structural part of glycosaminoglycans that constitute the cartilage, so ones it reaches the destination, its effectiveness is of no match thus delaying the progression of the disease^15^.

## Materials and methods

### Materials

#### Chemicals and reagents

Analytical grade Chloroform of Merck and analytical grade Methanol of Sigma Andrich were used as organic solvents for the formulation of transfersome and liposomes. Absolute Hydrochloric acid (Merck) and Sodium Hydroxide (Sigma-Aldrich, Germany) were used for the quantitative analysis of Glucosamine sulfate as per official compendia method. Triethylamine procured from Merck was used for the formulation of hydrogel. Eugenol was purchased from Sigma Scientific Pvt limited. Phosphate Buffer saline (PBS) solution having PH. 7.0 was made from PBS tablets purchased from Sigma Scientific. Distilled water was taken from pharmaceutics lab, Department of Pharmacy, Riphah International University (Islamabad, Pakistan).

For the formulation of transfersome, Phospholipid 90G was generously gifted by Lipoid Germany, Glucosamine sulfate was gifted from Polyline ChemPharma Peshawar, Polyoxyethylene (20) sorbitan monooleate (Tween 80) and sorbitan monostearate (Span 60) were procured from Merck. For the induction of osteoarthritis in knee joint of rabbits l-Cysteine and Papain were procured from Sigma Adrich Germany. All the chemicals were of pure analytical grade.

#### Apparatus and equipment

Rotary evaporator (Eyela), Weighing balance (Sartorius), PH meter, Hot plate multi stirrer, Pharmaceutical Refrigerator, Beakers, funnel, Titration apparatus, centrifuge machine, sonicator, test tubes.

### Methods

#### Synthetic procedure

##### Preparation of glucosamine sulphate loaded transfersomes

Glucosamine loaded Transfersomes were prepared by using thin film hydration method with minor modifications^16^. Briefly, phospholipid and surfactant in defined ratio were dissolved in 1:1 mixture of organic solvents (Chloroform: Methanol), and the resultant mixture was film dried in Rotary evaporator at temperature just above the phase transition temperature of phospholipid (i.e., 40 °C) and at reduced pressure in a tilt position to allow complete evaporation, removal of solvent leaving behind the thin film around the inner wall of flask. The thin film was allowed to dry overnight to allow complete removal of organic solvent. The Phosphate buffer saline solution was made with the help of PBS tablets. One tablet gave 100 ml of PBS solution of 7.5 pH when dissolved in distilled water. The PBS solution was divided into two portions. In one portion 7% absolute ethanol was added to produce 7%v/v PBS solution and other portion was kept as such with no addition of ethanol. The drug was dissolved in PBS and 7% ethanolic PBS solution in the concentration of 5%w/v. The drug solution was sprinkled with the help of syringe in the flask containing the thin film. The flask was rotated at 60 rpm for 1 h at 60 °C to allow the complete hydration of film. The hydration of thin film resulted in formation of Transfersomes. The resulting formulation was centrifuged at 4000 rpm to separate the unentrapped drug by removing the supernatant and remaining formulation was kept at 4 °C for further analysis.

##### Formulation of glucosamine loaded liposomes

Liposomes of GS were made by the conventional rotary evaporation method. Briefly phospholipid:cholesterol:surfactant was dissolved in 1:1 of organic solvents (Methanol:Chloroform), the resultant solution was evaporated in rotary evaporator under reduced pressure and temperature above phase transition temperature. The resultant dry film along the inner wall of flask was left overnight to allow complete removal of organic solvent. The film was then hydrated with 5% solution of GS in PBS solution by rotating at 60 for 1 h. The resultant solution of liposome was then bath sonicated to produce small unilaminar vesicles.

##### Optimization of transfersomes

2 factorial design was used to optimize the formulation variables. The independent variables were the type of surfactant used (either the Tween 80 or Span 80), the Phospholipid to Surfactant ratio and nature of PBS solution (either 7% ethanolic PBS or simple PBS solution). All the three independent variables were assessed by keeping the environmental conditions stable and constant for each formulation. The vesicle size of Transfersomes and entrapment efficacy was assessed as dependent variables.

10 different formulations were formulated as per below table (Table 1) and assessed for various physiochemical variables.

**Table 1:** Formulations of glucosamine sulphate loaded transfersome.

Composition, entrapment efficacy and vesicle size of Glu-loaded transfersomes					
Independent variables				Dependent variables	
Run	X1 (type of surfactant)	X2 (phospholipid:surfactant)	Hydration medium	Y1 (nm + SD)	Y2 (% + SD)
F1	Tween 80	80:20	7% ethanolic PBS solution		
F2	Span 80	80:20			
F3	Tween 80	90:10			
F4	Span 80	90:10			
S1	Span 80	90:10	PBS solution with 5% drug w/v		
S2	Span 80	85:15			
S3	Span 80	80:20			
S4	Tween 80	90:10			
S5	Tween 80	85:15			
S6	Tween 80	80:20			

where X1 = type of surfactant; X2: phospholipid: surfactant ratio; X3 = hydration medium used with 5% drug w/v; Y1 = vesicle size; Y2 = entrapment efficacy.

(a) *Vesicle size, PDI and zeta analysis* All the 10 formulations were characterized in term of entrapment efficacy, polydispersity index (PDI), zeta potential and mean vesicle size, PDI, zeta potential and mean vesicle size were determined by zeta sizer on request at Quaid-e-Azam University Islamabad. The 10 µl of transfersomal dispersion was diluted with distilled water 90 µl and sent for analysis in effendoff tube of 1.5 ml.

(b) *Entrapment efficiency analysis* Centrifugation method was used for the determination of entrapment efficacy. Briefly the prepared formulations were centrifuges at 4000 rpm for 15 min, resulting in sedimentation of vesicles leaving behind the unentrapped drug as a supernatant. The supernatant was collected and quantitatively analyzed for the glucosamine sulfate via titration method using 0.01 M sodium hydroxide as a titrant with phenolphthalein as an indicator. The appearance of light pink color was considered as an end point and 3.028 mg of Glucosamine sulfate utilized 1 ml of 0.01 M NAOH as per British pharmacopoeia. Entrapment efficiency was figured by using below formula^13^;

$${\text{EE}}\% = {\text{ A2}} - {\text{A1}}/{\text{A2 }} \times { 1}00$$

where A2 = total amount of drug added, A1 = drug un entrapped or un bound drug.

(c) *Surface morphology studies* The formulations were also analyzed for surface morphology using Scanning electron Microscopy (CRL lab University of Peshawar, Pakistan).

(d) *Deformability of glucosamine loaded transfersomes* The deformability was determined using the method as reported by^13^. Briefly transfersomal dispersion was extruded through 100 nm membrane filter (Filter-Bio^®^). After extrusion, the size of vesicles was analyzed along with weight of dispersion. The deformability index was then calculated by following formula^11^:

$$D=\mathrm{J}*\left(\frac{\mathrm{Rv}}{Rp}\right)^{2}.$$

where D = deformability index: J = weight of dispersion Rv = vesicle size after extrusion; Rp = pore size of filter.

#### Preparation of glucosamine loaded transfersomal gel

The prepared optimized formulation was not viscous enough to be applied trans dermally and also not stable enough to be stored for long period. That’s why the transformable dispersion was formulated as gel by using the method as reported by^13^. with minor modification. Briefly, transfersomes were added into 1% Carbopol 940 gel to make it rheological acceptable. 1 g of Carbopol 940 was added slowly with continuous stirring with machine to distilled water along with addition of transfersomal dispersion drop wise, to make volume up to 100 ml and triethanolamine was added slowly drop wise until a clear gel was formed. The PH of the resulting gel was analyzed by taking 1 g of gel and dissolving it in distilled water to make 50 ml, the triethanolamine was added until a neutral gel was obtained.

#### Physiochemical evaluation of glucosamine loaded transfersomal gel

The blank gel and the Glu-loaded transfersomal gel were evaluated in term of physical appearance, clarity, pH. Ph is determined by dissolving the 1-g gel in 50 ml of distilled water and measuring with the help of portable pH meter. Appearance and Clarity of gel were determined by placing the two formulations under the light of 400 lx.

#### Characterization of glucosamine loaded liposomes

The formed liposomal dispersion was analyzed for entrapment efficiency by centrifugation and zeta analysis by the same method as adopted for transfersomal dispersion.

#### Preparation of glucosamine liposomal gel

The gel was made by dissolving the vesicles in 1% w/w of Carbopol 940 and pH was made neutralized with the help of triethanolamine. And resulting gel was evaluated for pH, clarity and appearance by the same method as adopted for transfersomal gel^13^.

#### Physiochemical evaluation of liposomal gel

Resulting gel was evaluated for pH, clarity and appearance by the same method as adopted for transfersomal gel.

#### In vivo studies

##### Selection of animal

04 Healthy male rabbits, age 8 months were selected for in-vivo evaluation for effectiveness of therapy using glucosamine loaded transfersomes. Digital X-rays of all 4 rabbits knee joints were carried out at Pet and Vet clinic Islamabad. Moreover, radiological studies, CBC (Complete blood count) of rabbits were also performed to keep the data as a reference.

Rabbits were marked in numeric 1 to 4 using Cardinal Health Multi Ink Marker. 1st rabbit was kept as reference (control group), with no induction of osteoarthritis and treatment thereafter. All the remaining 3 rabbits, osteoarthritis was induced using the Bentley model and verified via radiological studies. The 2nd rabbit have been treated with liposomal formulation while 3rd and 4th been undergone treatment with transfersomal formulation for about 4 weeks.

Rabbits were kept in open space, fed with cereals, green vegetables, fruits with easy access to fresh drinking water, with no restriction to movement.

##### Preparation of papain: cysteine solution

For induction of osteoarthritis, Bentley model was adopted. By dissolving papain powder in water for injection (WFI) and co-administered along with cysteine solution to induce osteoarthritis in knee joint of rabbits, the solution of papain was prepared. Cysteine was added to act as activator^17^. pH of the resultant solution was checked.

##### Induction of osteoarthritis

Osteoarthritis was induced in 3 rabbits, with the average weight of 3 kg each. The initial x-rays were carried out at Pets and vet’s clinic Islamabad. The other 3 rabbits were injected using 25G B.Braun auto disable syringe with injection volume of papain: cysteine 0.2 ml: 0.1 ml at day 1. Injection was placed into the knee joint (intra-articular into the knee joint of right leg) of un-anesthetized rabbit avoiding the damage to the cartilage of the tibial plateau. The reference group (1st Rabbit) was injected with equal volume of normal saline solution. However, the left leg was not injected to allow free movement of rabbits. The same process was repeated on day 2.

After 6 days, the x-rays were carried out and show successful induction of osteoarthritis that is also visually apparent from movement of rabbits.

##### Application of formulated transfersomal and liposomal gel

After osteoarthritis development, as witnessed from the radiological studies, the rabbits were treated with transdermal application of prepared gel with mild rubbing. Previously the knee joints were shaved to sallow application of gel to the skin and avoiding sticking to the hairs. All the 3 rabbits were given the treatment initially for 2 weeks and x-rays were taken every week to access the healing process. Furthermore, the movement of rabbits were also noticed to witness the effect of gel. The second radiological studies were carried about after 2 weeks along with CBC analysis at pet and vet clinic Islamabad to record the observations.

##### Hematology and blood glucose studies

The blood samples were taken from all the rabbits prior to treatment with gel to determine the total blood count using the Medonic Hematology analyzer and blood glucose was determined by using Roche portable glucose meter. The same parameters were examined after the treatment to check the any change in blood composition after the application of drug.

## Results

### Formulation of glucosamine sulfate loaded transfersomes

Total of 10 formulations were made using thin film hydration method as per table (Table 2).

**Table 2:** Formulations using PBS with drug hydration medium.

2 × 2 factorial design (10 formulations were made)			
3 independent variables	02 dependent variables		
Type of surfactant (X1)	Entrapment efficacy		
Phospholipid:surfactant (X2)	Vesicle size		
Hydration medium			

Formulations			
Run	Type of surfactant (X1)	Phospholipid:surfactant (X2)	Hydration medium
F1	Tween 80	80:20	7% ethanolic PBS solution containing drug 5%
F2	Span 80	80:20	
F3	Tween 80	90:10	
F4	Span 80	90:10	
S1	Span 80	90:10	PBS solution containing drug 5%
S2	Span 80	85:15	
S3	Span 80	80:20	
S4	Tween 80	90:10	
S5	Tween 80	85:15	
S6	Tween 80	80:20	

6 formulations made using simple PBS solution were visually milky in texture while 4 formulations made using 7% ethanolic PBS solution tend to sediment soon after the 1 h cake formation occurred in formulations made using Span 80 as a surfactant, converting to homogeneous dispersion on shaking.

Thin film was successfully generated in all the cases and left overnight to allow complete remove of organic solvents, then hydrated with respective hydration medium while continuous stirring on the rotary evaporator.

### Entrapment efficiency

**Figure 1:**
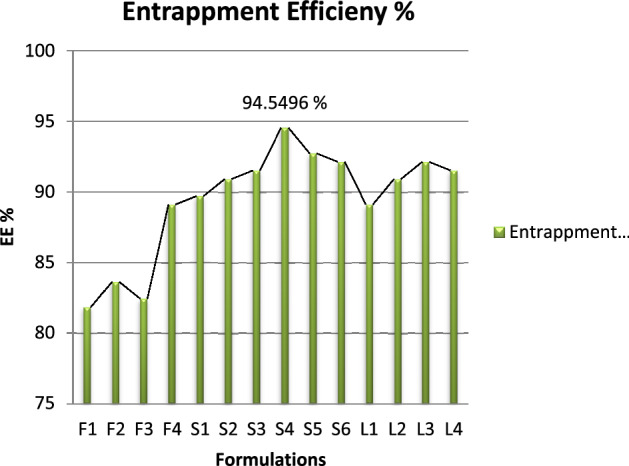
Graphical representation of EE results.

Entrapment efficiency of all the 10 formulations were recorded using the centrifugation method followed by titration method as per British Pharmacopeia shown in Table 3. Standard calibration curve of Glucosamine Sulphate was established by plotting concentration on x-axis vs volume used on y-axis as represented in Fig. 1. According to British Pharmacopeia, 3.028 mg uses 01 ml of 0.01 molar NaOH.

**Table 3:** Entrapment efficiency result.

Entrapment efficiency %	
Run	Entrapment efficiency %
F1	81.932
F2	83.6488
F3	82.4376
F4	89.0992
S1	89.7048
S2	90.916
S3	91.5216
S4	94.5496
S5	92.7328
S6	92.1272
L1	89.0992
L2	90.916
L3	92.1272
L4	91.5216

All the formulations were centrifuged at 4000 rpm for 15 min and supernatant was collected and analyzed for unentrapped drug.

### Vesicle size, PDI and zeta potential

**Figure 2:**
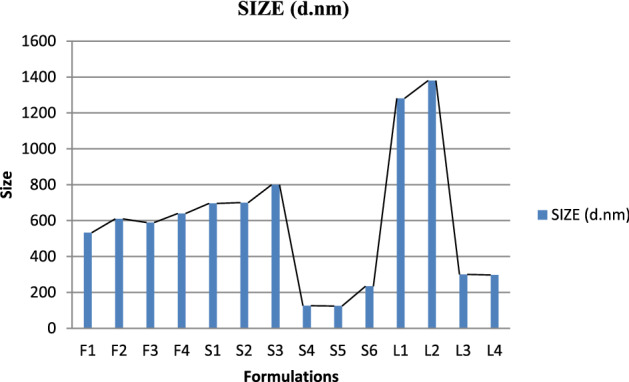
Graphical representation of vesicle size of Glu-loaded formulations.

**Figure 3:**
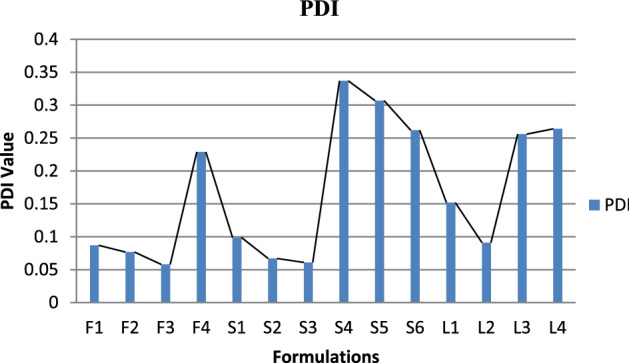
Graphical representation of PDI of Glu-loaded formulations.

**Figure 4:**
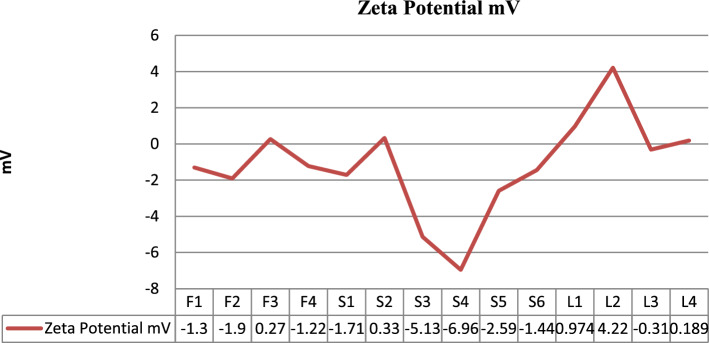
Graphical representation of zeta potential of Glu-loaded formulations.

PDI, zeta potential and mean vehicle size shown in Fig. 2 were get analyzed from Quaid-i-Azam university Islamabad tabulated below Table 4. The sample after dissolution were sent for analysis. PDI and zeta potential of Glu-Loaded formulations are displayed in Figs. 3 and 4 respectively. The following results were obtained:

**Table 4:** Results of size, PDI and zeta potential.

Run	Size (d nm)	PDI	Zeta potential mV
F1	532.6	0.087	− 1.3
F2	609.2	0.077	− 1.9
F3	589.4	0.058	0.27
F4	639.6	0.229	− 1.22
S1	696	0.099	− 1.71
S2	700.5	0.067	0.33
S3	800	0.061	− 5.13
S4	126	0.337	− 6.96
S5	**124.2**	**0.307**	**− 2.59**
S6	**234.3**	**0.262**	**− 1.44**
L1	**1281**	**0.152**	**0.974**
L2	**1380**	**0.091**	**4.22**
L3	**299.7**	**0.256**	**− 0.307**
L4	**297.2**	**0.264**	**0.189**

Significant values are given in bold.

### SEM analysis (surface morphology study)

**Figure 5:**
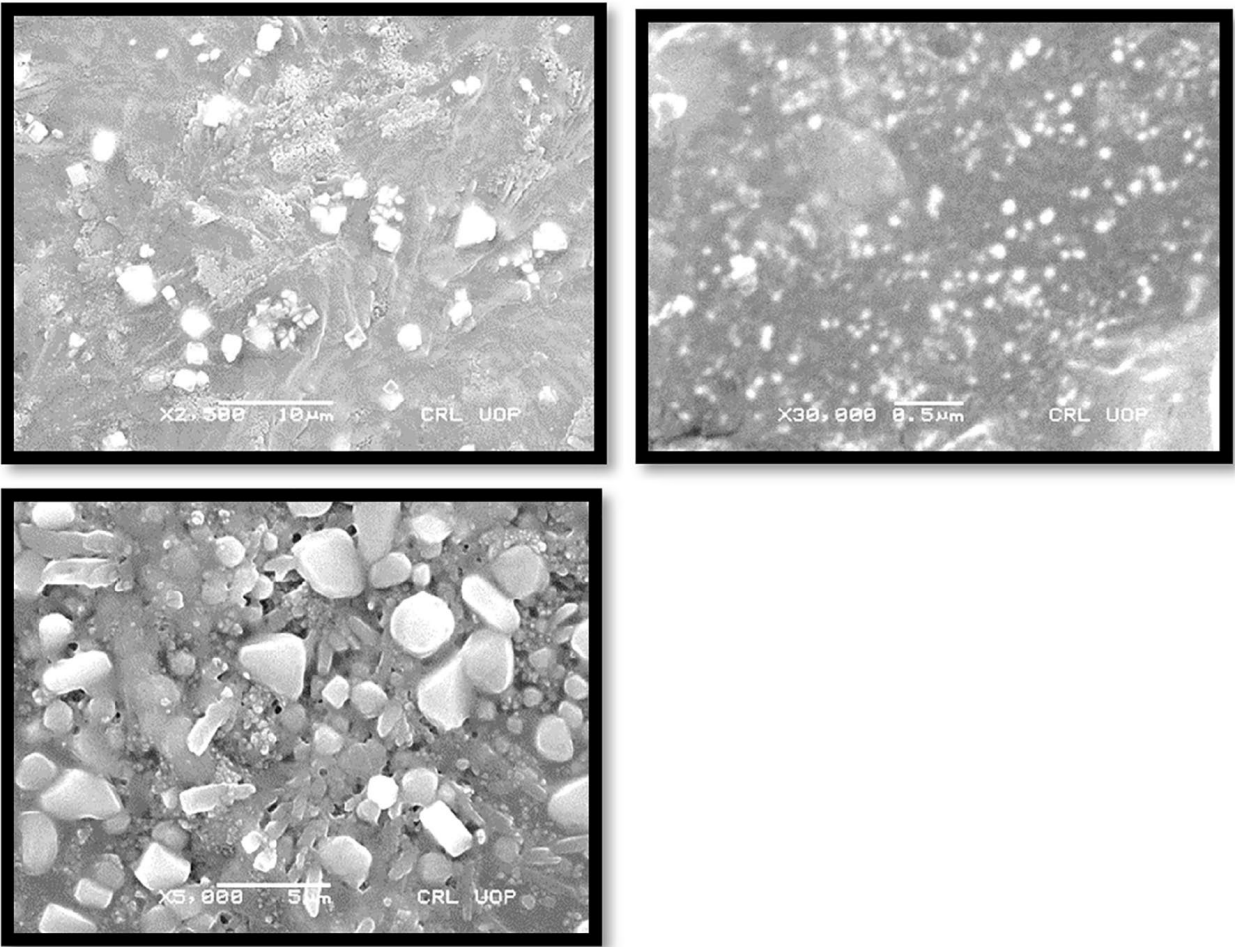
Microscopic evaluation of optimized Glu-loaded transfersome (S4 formulation).

**Figure 6:**
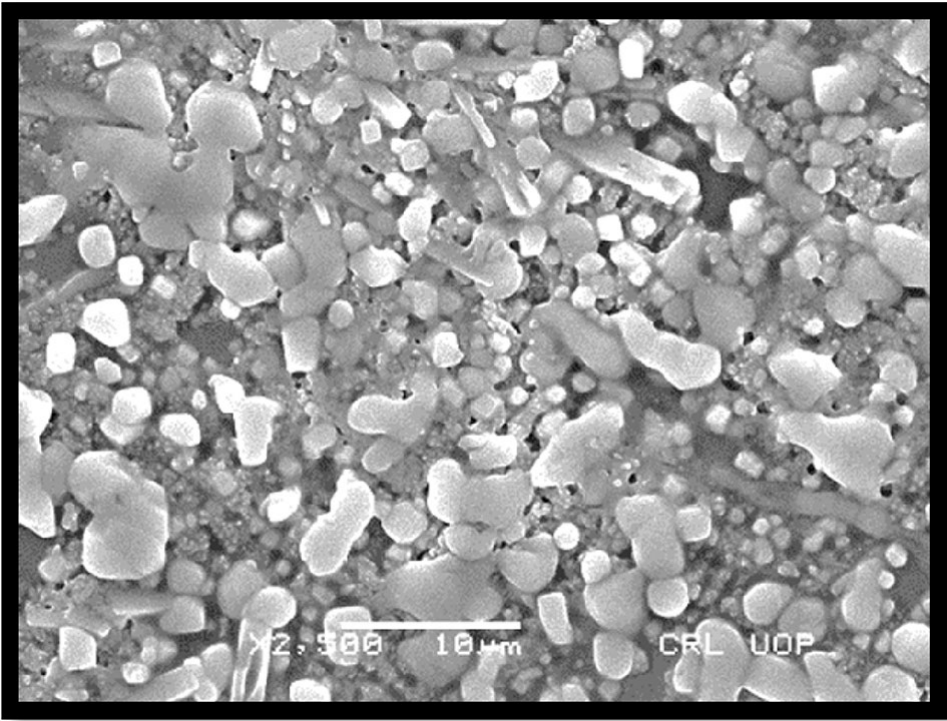
Microscopic evaluation of optimized Glu-loaded transfersome (S2 formulation).

Morphology of transfersomes was observed as shown in Fig. 5 using scanning electron microscope^18^ to determine the shape and lamellarity of transfersomes. Figure 6 shows Microscopic Evaluation of optimized Glu-Loaded Transfersome (S2 formulation) The following results were obtained:

### Deformability of glucosamine loaded transfersomes

DI (deformability index) of optimized formulation was calculated and tabulated in Table 5 using extrusion method, the following observations were observed:

**Table 5:** Deformability indices of S4 formulation.

Run	J (weight in grams of extruded dispersion)	Rv (vesicle size after extrusion)	Rp (pore size of filter in nm)	DI (Deformability Index in gram)
S4	7	125	100	10.9375
S4	8	124.2	100	12.340512

### Formulation of transfersomal gel

The optimized transfersomes were not viscous enough to stay on application site for a reasonable time. Therefore, the transfersomes were modified into gel as per method explained above. The pH was adjusted with triethanolamine to the neutral as presented in Table 6.

### Physiochemical evaluation of gel

**Table 6:** Physiochemical properties of gel.

Parameters	Physical appearance	Clarity	pH
Blank	Viscous, clear	Clear	7.1
Transfersomal loaded	Viscous, clear	Clear	7.1
Liposomal Loaded	Viscous, clear	Clear	7.0

### In vivo osteoarthritis healing capacity of the transfersomal gel

**Figure 7:**
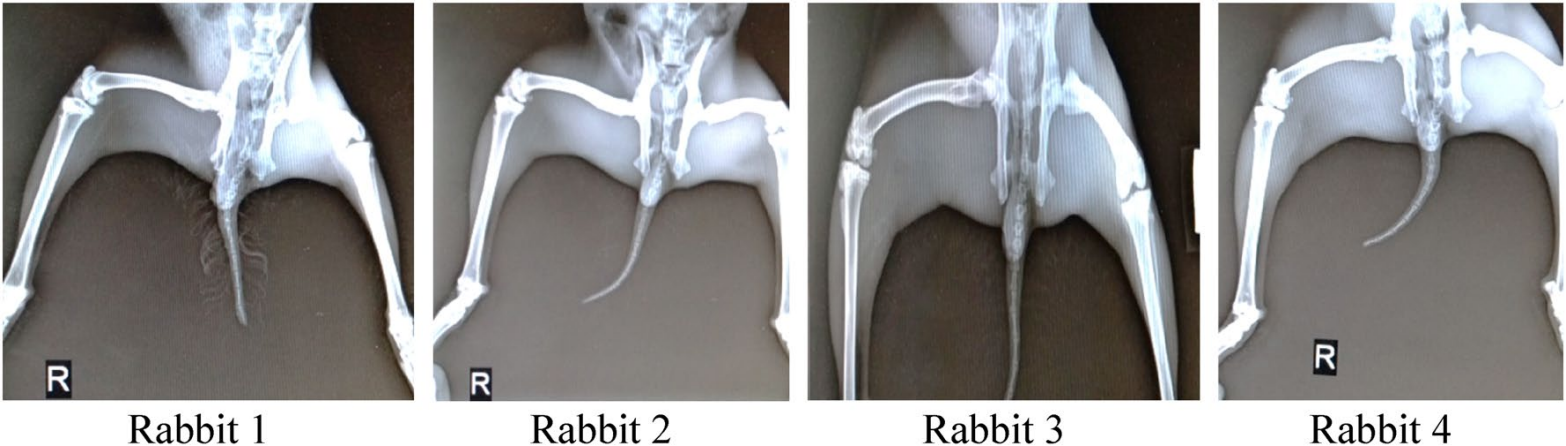
Initial x-rays of rabbits prior to induction of osteoarthritis.

4 rabbits were used in the test, initial radiological x-rays of both the knee joints were taken using Siemens Hybrid Digital X-ray machine and deformities are depicted in Fig. 7.

In addition to radiological study, CBC (complete blood count) using the Medonic Hematology Analyzer was also carried out along the blood sugar level detection using AccuChek Glucometer.

The following observations were found and expressed in Table 7:
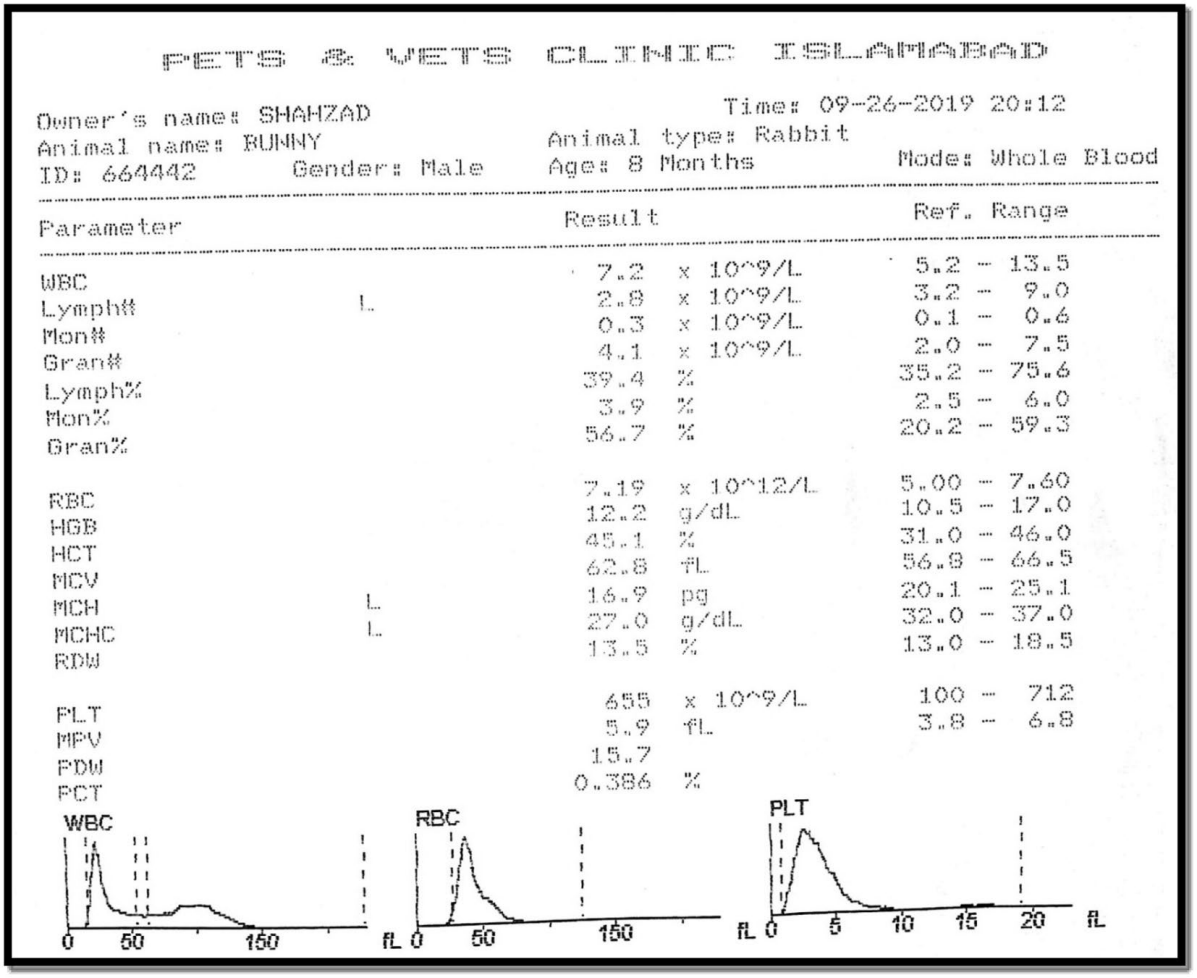


Blood glucose level was observed as follow:

**Table 7:** Blood glucose results.

Test animal	Device used	Day 1 (mg/dL)	Day 2 (mg/dL)
Rabbit 1		270	262
Rabbit 2		255	243
Rabbit 3		260	270
Rabbit 4		220	240

### Induction of osteoarthritis

4% solution of Papain and 0.03 M solution of Cysteine was prepared as per method described above and injected into right knee joint of 2nd, 3rd and 4th rabbit.

### Radiological and hematological results

**Figure 8:**
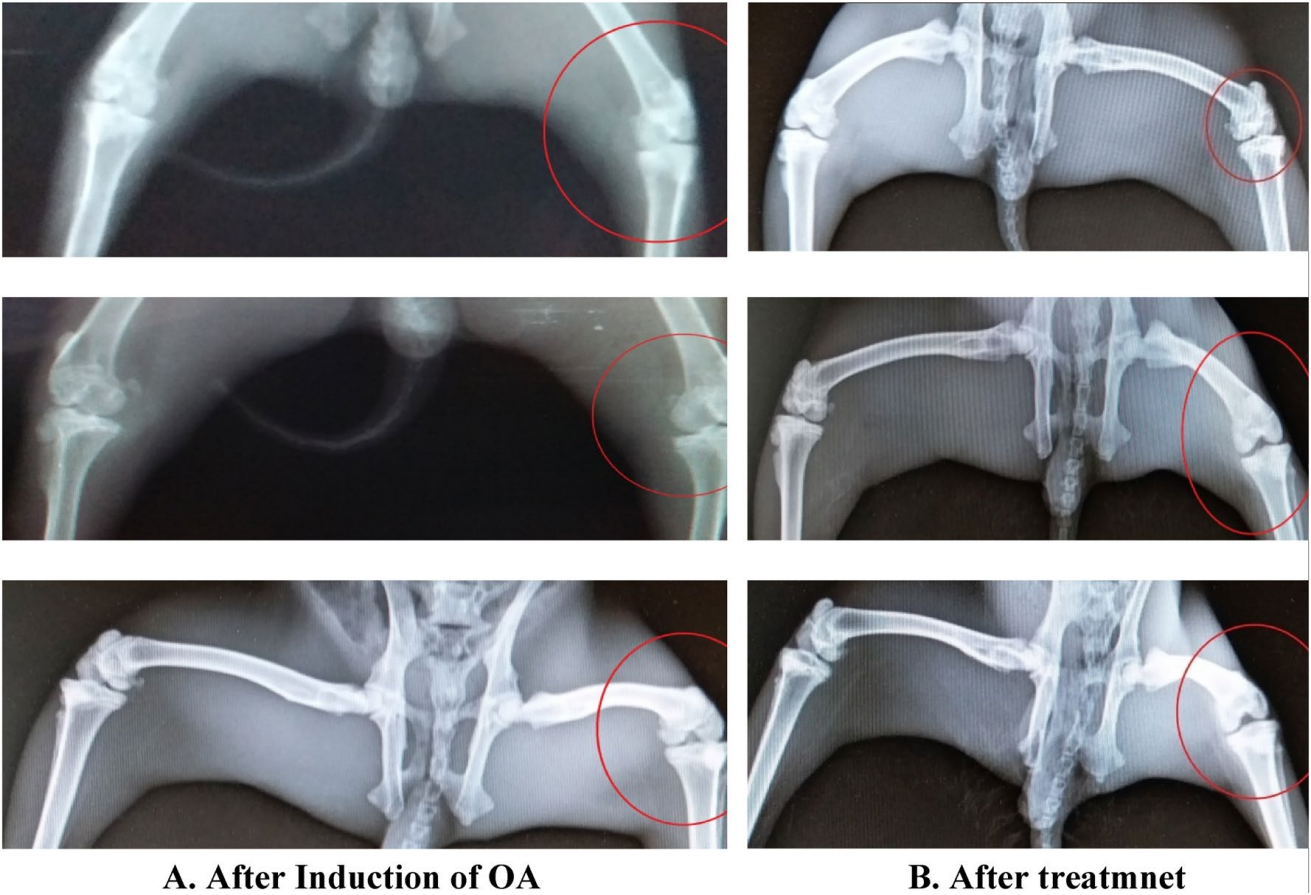
Radiological study results and observation.

The hematology studies shown in Fig. 8 were again carried out the application of drug for 1 month and upon good movement response by the rabbits except the one treated with liposomal gel. The following findings were observed in Table 8:

**Table 8:** Hematology and blood glucose results.

Test animal	Blood glucose (mg/dL)	CBC				
		WBC	LYMPH	RBC	PLT	MON
Rabbit 1	256	7.1	2.8	7.11	654	0.40
Rabbit 2	255	7.2	2.7	7.10	698	0.35
Rabbit 3	241	7.1	2.6	7.06	543	0.39
Rabbit 4	247	7.1	2.7	7.09	611	0.42

Reference range (as per pets and vets clinic Islamabad—Pakistan: WBC: 5.2–13.5, LYMPH: 3.2–9.0, RBC: 5.00–7.60, PLT: 100–712, MON: 0.1–0.6).

## Discussion

Osteoarthritis is a common disease of joints and it is affecting a large number of people worldwide. Osteoarthritis is characterized by progressive destruction of cartilage. Cartilage is smooth elastic tissue, composed of specialized cell called chondrocytes. Cartilage protects the ends of joints and provide a cushion for free movement of bones against each other. In disease condition, this cartilage gets damaged because of many reasons thereby exposing the underneath bone. The exposed bones rubbed against each other and generate pain, inflammation, difficulty in movement. Apparently, the OA is not diagnosable but predictable form symptoms feel by patient like the pain, swollen joint, difficulty in movement etc. Radiological studies help to diagnose the prevalence of disease. Various treatment includes exercise and use of pain killer to relieve the pain. Different Physiotherapy techniques also help to reduce the severity but not bringing the betterment in the integrity of damaged cartilage.

Cartilage is devoid of the access of blood vessels therefore restricting the bioavailibity of precursor’s molecules at the site of action. Treatment include the injection of various molecules with proven efficacy and safety in regeneration of cartilage. Studies have investigated the proven role of Glucosamine sulphate in the treatment of osteoarthritis as it is principal precursor of glycosaminoglycan, a major component of cartilage. But glucosamine sulphate has low bioavailability, also the synovial joint is avesicular and without any blood supply, thus limiting the effectiveness of traditional usage of glucosamine sulphate. Additionally, there have not yet been any studies focused on the development of Glucosamine sulphate transfersome. Therefore, we try to encapsulate the glucosamine sulphate into transfersome, which may have potential application for healing the degenerative disease of osteoarthritis.

Transfersomes are highly elastic and ultra-deformable vesicles, very similar to liposomes and act as carrier for transportation of various molecules. Due to elastic nature, they can easily cross the skin carrier and reach the site of action. So, transfersomes are selected to get incorporated with Glucosamine sulphate, to allow painless and easy availability of salt at the site of action to initiate the process of cartilage regeneration. Glucosamine sulphate is an amino sugar with molar mass of 179.17 g/mol and supplemental glucosamine may help to rebuild cartilage and treat arthritis.

In this study, we successfully prepared transfersome using thin film hydration method. This method of preparation was chosen keeping in view the availability of required apparatus/equipment (Rotary Evaporator) and it is the most effective method of formulating transfersomes in which thin film is prepared hydrated and then brought to the desired size by sonication. All the preparation was carried out at controlled lab environmental conditions. Concluding the tested parameters, the best production condition was operating the rotary evaporator at the water bath temperature of 45 degree Celsius with 250 ml round bottom rotary flask at the angle 45 degree with the RPM of 60. Operating the apparatus at less than 60 RPM and below temperature of 45 degree Celsius resulted in gel like mass at the center of rotary flask, that is the mixture of phospholipid and surfactant due to poor evaporation of organic solvent.

The optimized formulation was 5% drug loaded in 90:10 ratio of Phospholipid: Tween 80 with hydration with PBS solution. Use of Ethanolic PBS solution didn’t result in satisfactory production of transfersome and resulted in sedimentation of vesicles. This might be due to rupturing of vesicles and non-entrapment of drug which settle at the bottom on the tube. Furthermore, these conditions were also used to compare the other edge activator i.e., Span 80.

Span 80 was also used to formulate different test formulation of glucosamine loaded transfersomes. The formulations made with Span-80 trend to be larger in vesicle size (with average of 750 nm) and also lead to creaming i.e., the formed vesicles get accumulated at the top of tube leaving below a clear liquid layer. According to the evaluation of particle size, entrapment efficiency and zeta potential, the optimal particle size of S4 formulation was 126 nm with entrapment efficiency 94.54% calculated using official compendial method. However, the zeta potential was not significantly good enough to claim the stable production of transfersomes. Therefore, the vesicles were entrapped in secondary gel of Carbopol 940. The formation of gel resulted in stable formulation with clear appearance, neutral pH and better viscosity to allow better application of skin. The transfersomal formulation was added drop wise near the shaft of mixer, avoiding the direct contact with shaft and dropping from too much height.

Scanning Electron Microscopy suggested the morphology of transfersome having pretty satisfactory shape and size to allow the penetration of transfersome across the skin barrier. The deformability index of optimized formulation was found good having the value of 10–12 g. The availability of drug at the site of action is the most desirable attribute of this study that was assessed by analyzing the radiological studies and blood glucose level of rabbits. Male healthy albino rabbits of 8 months age and approximately 3 kg weight were used in the study, feed on natural diet and without restricted movement.

In the in vivo analysis, osteoarthritis was first induced in the 3 rabbits following the Bentley Model and development of osteoarthritis was confirmed from radiological studies. The induction of osteoarthritis was also clearly visible from movements of rabbits. The rabbits with injections of papain: cysteine have less movement compared to the reference rabbit and they also fold the leg with injection to cop pain and osteoarthritis. After the induction of osteoarthritis, the effectiveness of prepared formulations was analyzed. The formulated drug in gel form was applied with mild rubbing and rabbits are left free to move. After one month of regular application of drug formulations, the 2 rabbits showed better regeneration of cartilage without any significant changes in the CBC (Complete blood count). However, the test animal treated with liposomal formulation failed to show any improvement. The treatment was kept continue for one month with periodic checks on radiological and hematological results. One of the rabbits is also treated with Liposomal formulation, didn’t show any improvement in osteoarthritis. That might be due to non-penetrability of liposomal formulation across the skin barrier.

Therefore, this result also indicated that the application of transfersome in the future might improve the bioavailability in many respects however the stability of the transfersomal formulation is an issue that can be overcome using the combination of different edge activators.

## Conclusion

Transfersomes are proven the better drug delivery through the management and treatment of osteoarthritis. Glucosamine sulphate have a vital role in the treatment of osteoarthritis but effectiveness is restricted due to un-availability of drug at the site of action. Glucosamine sulphate is the principal precursor of glycosaminoglycan, which is the major component of cartilage.

Nano deformable transfersomes of Glucosamine sulphate were successfully formulated, optimized, physiochemically characterized and in vivo evaluated to support the ability of transfersomes in treatment and management of osteoarthritis. Transfersomal gel was formulated to increase the stability of vesicles carrying the drug and for easily application on skin.

The results obtained as per radiological study of rabbit’s knee joint strongly support the effectiveness and usefulness of transfersomes for the transdermal delivery of drug. The study helps to develop similar formulations for the management and treatment of various diseases, where the bioavailability of drug at target site is the issue.

The only limitations associated with this study is the shelf life of the prepared formulations which is not assessed for final gel formulation. By changing various parameters, it might be possible to obtain more stable formulation and real time assessment of stability study can bring further improvement in the study.

## Data Availability

All data relevant to the study are included in the article or no data is uploaded as supplementary information.
